# The ageing body: contributing attitudinal factors towards perceptual body size estimates in younger and middle-aged women

**DOI:** 10.1007/s00737-020-01046-8

**Published:** 2020-06-19

**Authors:** Ashleigh M. Bellard, Piers L. Cornelissen, Emanuel Mian, Valentina Cazzato

**Affiliations:** 1 School of Psychology, Faculty of Health, Liverpool John Moores University, Liverpool, UK; 2 Department of Psychology, Northumbria University, Newcastle, UK; 3 Emotifood Body Image & Eating Disorders Unit, 20900 Monza, Italy

**Keywords:** Attitudinal, Perceptual, Body image, Body size estimates, Middle-aged, BMI

## Abstract

Over-estimation of body size, a core feature of eating disorders (EDs), has been well-documented both in young healthy and ED individuals. Yet, evidence that altered body perception might also affect older women is limited. Here, we examined whether attitudinal components of body image (i.e. the feelings an individual has about their body size and shape) might affect perceived actual and ideal body shape self-estimates in midlife, similarly to younger women. Thirty-two younger (mean age, 24.22 years) and 33 middle-aged (mean age, 53.79 years) women took part to a computerized body perception assessment of perceived, actual and ideal aspects of body image. Body mass index (BMI), societal and interpersonal aspects of appearance ideals, measured by means of Sociocultural Attitudes towards Appearance Questionnaire (SATAQ-4), and assessment of body uneasiness and concerns for specific body parts, measured by Body Uneasiness Test (BUT-A/B) scales, were also investigated. Younger and middle-aged women with larger BMI showed greater discrepancy in perceptual distortions from their perceived actual body size. However, middle-aged women with greater body part concerns overestimated their perceived body size, as opposed to younger women who were almost accurate. Unlike middle-aged women, younger women with higher body part concerns desired slimmer ideal body image than their perceived actual. Results suggest that distortions in the perceived actual and ideal body size self-estimates of younger and middle-aged women are best explained by a combination of BMI, body part concerns and the particular age group to which a participant belonged. In the future, a personalized approach for the assessment of women’s perceptions and concerns of specific body areas during lifespan should be adopted.

**Electronic supplementary material:**

The online version of this article (10.1007/s00737-020-01046-8) contains supplementary material, which is available to authorized users.

## Introduction

Body image is a multidimensional construct that represents an individual’s conscious perception of, and attitude towards, their bodily appearance (Arbour and Ginis 2008; Reboussin et al. 2000; Tiggemann 2004). It is strongly associated with an individual’s wellbeing and their satisfaction with life (Donaghue 2009). According to an influential meta-analysis by Cash and Deagle (1997), dimensions relevant to the body image construct substantially include the following: (i) a perceptual component which corresponds to the accuracy with which an individual can judge the physical dimensions of their own body and (ii) an attitudinal, affective component related to their attitudes and emotions they have about their body, which may be positive or negative.

Excessive concerns about body weight and appearance are common amongst individuals in western cultures and presage the development of eating disorders (EDs), such as anorexia nervosa (AN) and bulimia nervosa (Ricciardelli and McCabe 2004; Slevec and Tiggemann 2011a; Stice 2002). Typically, individuals with severe body image concerns focus on the desire to appear thinner (Slevec and Tiggemann 2011a) and tend to overestimate the body size they believe themselves to have (Schuck et al. 2018).

Most research into people’s body image concerns and eating attitudes has been conducted in adolescents and young adults, with a particular focus on female university undergraduates aged 18–24 years (Slevec and Tiggemann 2011b). This coincides with the median age of onset for EDs (Hudson et al. 2007; Favaro et al. 2003), with AN being particularly prevalent in this age group (Smink et al. 2012). Nevertheless, as pointed out by Saucier (2004) and Tiggemann (2004), comparably high levels of body image concerns may occur at any age, including women of middle-age who represent the focus for the current study. As with their younger colleagues (Wardle et al. 2006), middle-aged women can experience negative feelings and attitudes towards their body, such as body dissatisfaction and drive for thinness (Bane and McAuley 1998; Longo et al. 2009) along with over-estimation of their body size (Hayashi et al. 2006; but see Monteath and McCabe 1997 and Paul et al. 2015 for opposite results in the general population) leading to an increased risk for late onset EDs (Marcus et al. 2007; Cumella and Kally 2008; Hoek 2006; Slevec and Tiggemann 2011a).

However, unlike young women, some of the factors giving rise to distorted body image in middle-age emerge from naturally occurring age-related processes. These include an increase in body weight/fat distribution due to the menopause and a decrease in muscle mass, which may also be side effects of different medications (Davis et al. 2012; Genazzani and Gambacciani 2006; Tchkonia et al. 2010; Vanina et al. 2002). As a result of these natural occurring age-related changes in their body shape, middle-aged women may exert more of a need for slimmer weight control to be classed as ‘attractive’, as they still show attentiveness towards their bodily appearance (Lewis and Cachelin 2001; McCabe et al. 2007; Pruis and Janowsky 2010). Linked to the need to maintain a thinner body size, Marcus et al. (2007) have identified an increase in the number of middle-aged women being diagnosed with an ED, i.e. 175 out of 589 middle-aged women of various ethnicities reported having an ED, predominately AN.

Furthermore, although several studies suggest that body image in younger women is more susceptible to societal influence to attain a slim physique than in older women (Lewis and Cachelin 2001), others reported that societal influence is also a predictor of body dissatisfaction, drive for thinness and body shape concerns in older women (Pruis and Janowsky 2010). This suggests that societal influence is an important factor in the development and maintenance of negative attitudes towards body image of older women and that it may be pertinent to understanding body image in this age group.

Put together, findings like these suggest the need for further research specifically targeting middle-aged women. By shedding light on the perceptual mechanisms and women’s attitudes and feelings towards their body shape, it might be possible to provide further insight into the predictive factors that may trigger, maintain and exacerbate symptoms in those psychiatric conditions characterized by body image disturbances (e.g. EDs and body dysmorphic disorders) and hopefully contribute to the development of novel individualized body image treatments in lifespan.

## The current study

In this study, we aimed at investigating whether performance in tasks that measure perceptual (i.e. participants’ estimates of their own body shape) or attitudinal aspects (i.e. feelings and attitudes towards body shape and size) of body image that are relevant to the development of ED symptomatology are essentially the same or different in younger compared with middle-aged women. With this aim, in separate samples of younger and middle-aged women, we assessed perceptual self-estimates of perceived current (i.e. ‘How do you think you look like?’) and ideal (i.e. ‘How would you like to appear?’) body shapes, by means of a unique 2D digital computer-based distortion optical method, the Body Image Revealer (BIR, Mian and Gerbino 2009). In addition, we obtained measures of women’s attitudinal body image, by means of a battery of standard self-report scales to index women’s feelings and attitudes towards their body shape and beauty ideals. With these regards, we felt that it was important to have a wider range of self-report scales than has often been the case in similar studies of younger women. For example, in three such studies, Cornelissen and colleagues used the Beck Depression Inventory, the Rosenberg Self Esteem scale, the Body Shape Questionnaire and the Eating Disorder Examination Questionnaire to measure the participants’ attitudes to body shape, weight, eating, self-esteem and depressive symptomatology (Cornelissen et al. 2015, 2017; Irvine et al. 2019). In each study, a principal component analysis (PCA) of the psychometric responses showed that the data could be compressed onto a *single* principal component, or dimension, suggesting a rather restricted view of participants’ attitudinal body image. Therefore, here we chose a wider spectrum of measurement including a measure of the cultural and interpersonal risk factors, such as internalization of appearance ideals and appearance-related pressures which have been implicated in the aetiology of negative body image and eating pathology (Cafri et al. 2005; Stice 2002), here assessed by means of the Sociocultural Attitudes Towards Appearance Questionnaire-4 (SATAQ-4, Schaefer et al. 2015). Furthermore, given that healthy and clinical populations often report great dissatisfaction with body areas like weight and torso (lower, mid and upper) (see Rosen and Ramirez 1998; Hrabosky et al. 2009), but also buttocks/hips/thighs, stomach and waist regions (Toh et al. 2019; Ralph-Nearman et al. 2019), we administered a measure of body uneasiness and dissatisfaction for the whole body and for specific body parts, by means of the Body Uneasiness Test (BUT, Cuzzolaro et al. 2006). Anthropometric measures of body mass index (BMI) were also measured. Finally, we applied a multivariate analysis to investigate how similar was the pattern of responses across the two age groups.

Consistent with the view of a multidimensional model of body image (Cash and Deagle, 1997), we expected that perceptual self-estimates of perceived current and ideal body shape should best be predicted by a combination of participants own’ BMI and their attitudes and feelings towards their body shape/body parts, as well as internalization of beauty ideals. In agreement with Pruis and Janowsky’ results (Pruis and Janowsky 2010) which provided evidence that ratings of body image do not differ in normal, healthy younger and older women when personalized measures of body shape assessment (in their study women’s responses to line drawings of bodies in the Figure Ratings Scale) are used, we also expected that BMI and body shape concerns would be predictive of women’s perceptual body size estimates in a way that should be similar in both age groups. However, consistent with studies suggesting stronger societal influence on body image in younger than older women, particularly pressure to conform to the media ideal of women’s bodies (Bedford and Johnson 2006; Lewis and Cachelin 2001), we expected younger women’s perceptual body size estimates to be more influenced by their levels of societal influence and pressures to attain a slim physique, compared with older women.

## Materials and methods

### Participants

Sample size calculation was based on the data from Irvine et al. (2019). In this study, 100 healthy adult women carried out a number of tasks including a psychophysical procedure for self-estimation of body size; they had their BMI measured, and they carried out the body shape questionnaire (BSQ; Evans and Dolan 1993). First, we calculated multiple regression analyses in which body size self-estimation was predicted from a combination of BMI and BSQ. Then, we used PROC POWER in SAS v9.4 (SAS Institute, North Carolina, USA) to calculate sample sizes appropriate to estimate the effects of BMI and, separately, BSQ, at an alpha value = 0.01 and a power = 0.8. This rendered integer sample sizes for BMI and BSQ of 23 and 59 respectively. To offset attrition in participant numbers and/or unexpected sources of variability, we therefore recruited a total of 65 females (as assigned at birth) to the study who gave their written consent to take part.

Participants, who self-identified as Caucasian, were preselected and assigned to two groups based upon age: 32 participants were recruited to the younger women’s group (age *M* = 24.22 years; *SD* = 4.51 years; range, 18–37 years) and 33 participants were recruited to the middle-aged women’s group (age *M* = 53.79 years; *SD* = 3.72 years; range, 47–65 years; see Table 2). All participants were recruited externally through poster advertisements situated in public locations, through social media and through individuals known to the researcher. Younger women were also recruited internally through the Liverpool John Moores University (LJMU) Psychology SONA participation scheme for undergraduate Psychology students. Middle-aged women were also recruited internally through members of staff at LJMU. Furthermore, some middle-aged women that had been in prior lab studies (unrelated to body image) were contacted from our database of previous study participants (Psychology Research Participants Panel). All participants were provided with an information sheet prior to investigation, in order to check for eligibility based on the study inclusion criteria, which was also confirmed on the day of the experiment. Participants were only eligible to take part if they (self)reported not to have any history of neurological or psychiatric disorders, including EDs, had normal or corrected visual acuity and were not pregnant. As an incentive, participants either received SONA (participation point scheme) points (if undergraduate students) and/or £10 in shopping vouchers. Younger women’s BMIs ranged between 17.73 and 33.18 (M = 22.74, SD = 4.36) and fell into the following WHO categories: 4 underweight, 20 normal, 5 overweight and 3 obese. Middle-aged women’s BMIs ranged between 18.93 and 38.83 (M = 27.16, SD = 5.13) and fell into the following WHO categories: 15 normal, 7 overweight, 7 obese and 4 severely obese. The study’s experimental procedures and methods were fully approved by LJMU Research Ethics Board and complied with the ethical standards of the 1964 Declaration of Helsinki.

### Assessment of body image

#### Sociocultural Attitudes towards Appearance Questionnaire

The Sociocultural Attitudes towards Appearance Questionnaire-4 (SATAQ-4, Thompson et al. 2004) measures a woman’s drive to attain attractiveness ideals dictated by societal influence (Schaefer et al. 2015). For SATAQ-4, participants evaluated each of the 22 items on a 5-point scale (from 1 = definitely disagree to 5 = definitely agree). The questionnaire comprises of 4 subscales: internalization athletic, internalization body fat, pressures from family, pressures from peers and pressures from the media (Thompson et al. 2004). This questionnaire had good internal consistency with Cronbach’s alpha 0.81.

#### Body Uneasiness Test

The Body Uneasiness Test (Cuzzolaro et al. 2006) is considered a valuable tool for the screening and for the clinical assessment of abnormal body image attitudes and EDs. Particularly, it assesses body uneasiness and dissatisfaction with the whole body, as well as specific body parts. It comprises of 34 questions about body experiences (BUT-A) and 37 questions about an individual’s dislike of particular body parts (BUT-B). BUT-A is divided into 5 subscales: weight phobia (BUT-WP), dissatisfaction regarding the body and its weight, body image concerns (BUT-BIC), avoiding and compulsive self-monitoring behaviour (BUT-AV, BUT-CSM) and experience of depersonalization, defined as separation and foreignness regarding the body (BUT-D). These scores can be combined into a Global Severity Index (GSI, the average rating of all 34 items constituting the BUT-A), which indicates severity of abnormal body image concerns and eating behaviours. Each question is indexed by a 6-point Likert scale, from 0 representing ‘never’ to 5 representing ‘always’. Higher scores indicate greater body uneasiness.

BUT-B measures specific worries about particular body parts, shapes or functions (e.g. mouth or skin). These scores are arranged into a Positive Symptom Total (PST, the number of symptoms rated higher than zero) and a Positive Distress Symptom Index (PDSI, the average rating of those items constituting the PST). A 6-point Likert scale, which ranges from 0 (never) to 5 (always) indicating how often participants happen to dislike each experience or part of their body, is used. The Body Uneasiness Test showed good internal consistency with Cronbach’s alpha 0.90.

#### Body mass index

Each participant’s actual body mass index (BMI) was physically measured and calculated from their weight and height by using a calibrated bioimpedance digital scale (OMRON BF511) for weight and a stadiometer for height.

#### Body Image Revealer

Perceived actual and ideal body size estimates were obtained by means of a computer-based method, which mimics changes in adiposity by simulating an optical distortion of the body. Known as the ‘Body Image Revealer’ (BIR; Mian and Gerbino 2009; Cazzato et al. 2015, 2016; Zamariola et al. 2017), this technique provides a measure of the discrepancy between the dimensions of the real image and the sizes attributed by the participant during the task. The BIR has good ecological validity because it gives participants the experience equivalent to looking at their whole body in a mirror.

**Fig. 1:**
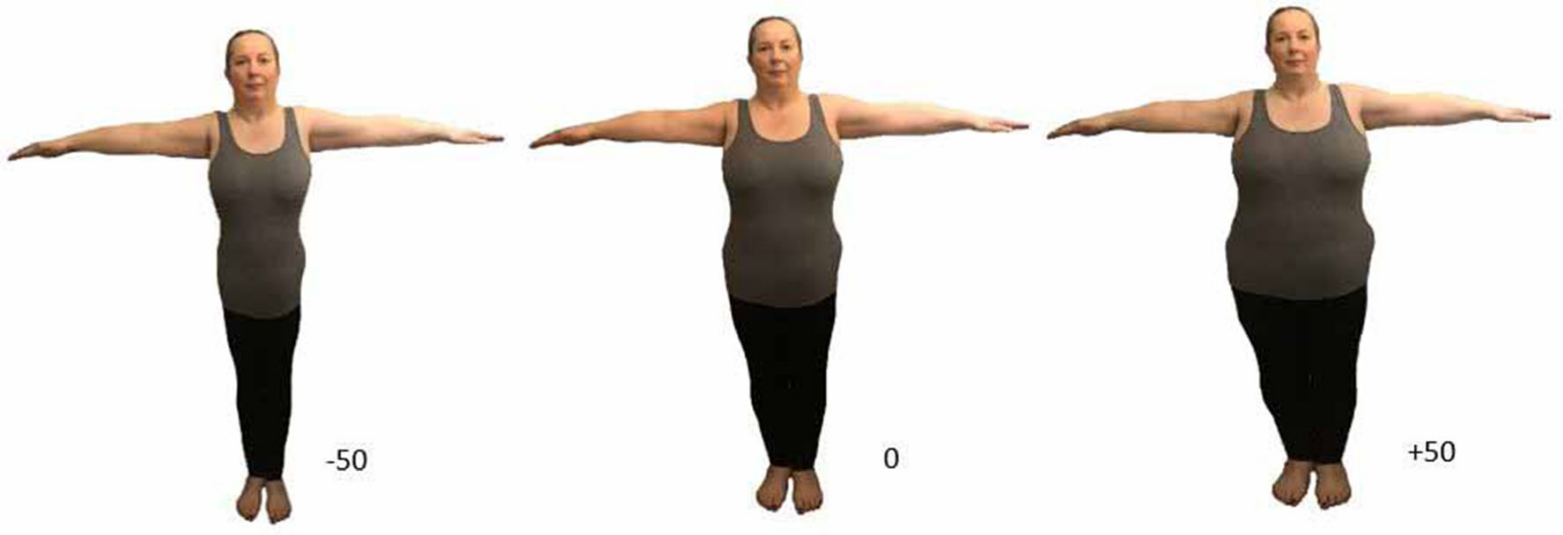
Visual representation of the body distortion technique, using the Body Image Revealer (BIR); veridical (0 = original, centre), distortion (− 50 = slimmer and + 50 = fatter) of body size. Images of participants were viewed against a white background

To generate the experimental stimuli, a frontal picture of each participant, standing in a T-pose, was taken with a Panasonic TZ5 Lumix digital camera from a distance of 2 m. Participants wore skintight clothing to ensure that their body outline was clearly visible. The image of a participant’s was then extracted from the background in the raw image, using Photoshop v7.0, and replaced on a standard white background for further image manipulation in BIR. Once modified, the image was opened in the software, and the experimenter selected the parts that would be modified, that is, from the neck to the feet (but excluding the face and the arms). Importantly, whilst the experimenter was modifying the real picture, participants were instructed to look away from the PC monitor, so they were not aware of the body alteration. Importantly, the ecological validity of the test was increased by keeping the participants’ face in the final images. This way, the procedure was giving the sense that participants were looking at themselves in the mirror (see Fig. 1 and Online Resource 1 for more details).

#### Procedure

During the experimental session, consenting participants’ height and weight were physically measured by using a calibrated bioimpedance digital scale and a stadiometer and then their portrait taken. Whilst this image was being edited, participants completed the demographic questionnaires. Once completed, participants were sat 55 cm in front of the display monitor and were asked to adjust their image according to two tasks read out to them: perceived actual body image (‘How do you think you look like?’) and ideal body image (‘How would you like to appear?’). By pressing the plus (+) or minus (−) key on the keyboard, participants were able to increase or decrease the apparent adiposity of the image within a possible range of ± 50% in 1% increments/decrements (see Online Resource 1). Participants could adjust the degree of distortion of the picture as much as they wanted. After completion of the two self-body distortion tasks, participants were instructed to fill out the BUT-A/B and SATAQ-4 questionnaires. Overall, testing lasted 45 min.

#### Statistical analyses

All statistical analyses were conducted using SAS v9.4 (SAS Institute, North Carolina, USA). In keeping with previous studies (Cazzato et al. 2014, 2016), the average percentage body percentage distortion (%BDS) was calculated across all trials, separately for each individual and the two tasks (i.e. perceived actual body size, ideal body size).

We wanted to model the relationships between participants’ estimates of their perceived actual and ideal body size predicted from participants’ AGE. In addition, we wanted to control for any influence of BMI and the psychometric variables (BUT-A/B and SATAQ-4). In order to avoid the possibility of introducing substantial variance inflation into the models, we first checked for evidence of co-linearity amongst the psychometric variables.

We used PROC CORR in SAS v9.4 to compute Pearson’s correlations between all self-report psychometric task subscales, to look for potential association within and between the responses to the BUT-A, BUT-B and SATAQ-4. Given that this analysis demonstrated substantial correlations amongst these variables (see Table 1), we then used PROC FACTOR in SAS v9.4 to carry out a PCA on this correlation matrix, to identify the smallest number of statistically independent dimensions in the psychometric tasks that we could use as covariates in our multivariate analysis and to avoid variance inflation due to multicollinearity amongst explanatory variables. We found four components, corresponding to the following: (i) the body part responses in the BUT (referred to henceforth as BUT-Parts); (ii) attitudinal responses in the BUT (referred to henceforth as BUT-Att); (iii) responses related to social pressure from the SATAQ-4 (referred to henceforth as SATAQ-Press); and (iv) responses related to internalization from the SATAQ-4(referred to henceforth as SATAQ-Int) (see Online Resource 2).

**Table 1:** Pearson’s correlation coefficients for the inter-correlations for BMI and the 18 subscales of the self-report questionnaires for both young and middle-aged women combined

	1	2	3	4	5	6	7	8	9	10	11	12	13	14	15	16	17	18
1 BMI	-																	
2 SATAQ_INTT	0.14	-																
3 SATAQ_INTM	− 0.14	0.54***	-															
4 SATAQ_FP	0.50***	0.32**	0.05	-														
5 SATAQ_PP	0.36**	0.45**	0.37**	0.63***	-													
6 SATAQ_PM	0.40**	0.24*	0.09	0.33**	0.31*	-												
7 BUT_BIC	0.67***	0.40**	0.11	0.40**	0.39**	0.50***	-											
8 BUT_A	0.59***	0.32**	0.06	0.35**	0.38**	0.33**	0.84***	-										
9 BUT_CSM	0.28*	0.52***	0.38**	0.24	0.45**	0.39**	0.70***	0.61***	-									
10 BUT_D	0.27*	0.41**	0.31*	0.11	0.29*	0.18	0.58***	0.64***	0.76***	-								
11 BUT_WP	0.54***	0.41**	0.10	0.25*	0.36**	0.51***	0.88***	0.76***	0.81***	0.71***	-							
12 BUT_M	0.22	0.42**	0.35**	0.20	0.31*	0.28*	0.52***	0.43**	0.58***	0.47***	0.54***	-						
13 BUT_FS	0.35**	0.37**	0.16	0.30*	0.31**	0.29*	0.58***	0.51***	0.62***	0.56***	0.65***	0.66***	-					
14 BUT_TH	0.67***	0.24	− 0.04	0.41**	0.32*	0.44**	0.69***	0.58***	0.52***	0.37**	0.67***	0.52***	0.62***	-				
15 BUT_L	0.40**	0.40**	0.18	0.21	0.27*	0.41**	0.61***	0.47***	0.62***	0.50***	0.69***	0.70***	0.70***	0.71***	-			
16 BUT_H	0.34**	0.33**	0.04	0.25*	0.25	0.37**	0.62***	0.55***	0.59***	0.44**	0.69***	0.73***	0.80***	0.66***	0.78***	-		
17 BUT_MOU	0.23	0.23	0.07	0.08	0.15	0.15	0.41**	0.30*	0.49***	0.36**	0.45**	0.38**	0.52***	0.35**	0.44**	0.43**	-	
18 BUT_SK	0.16	0.35**	0.26*	0.15	0.39**	0.25*	0.50***	0.41**	0.66***	0.56***	0.56***	0.57***	0.63***	0.47***	0.55***	0.57***	0.37**	-
19 BUT_BLU	0.31*	0.30*	0.22	0.31*	0.33**	0.27*	0.45**	0.40**	0.56***	0.50***	0.52***	0.52***	0.72***	0.52***	0.58***	0.64***	0.51***	0.52***

*p < 0.05, **p < 0.01, ***p < 0.0001

*BMI* body mass index, *SATAQ* Sociocultural Attitudes Towards Appearance Questionnaire, *BUT* Body Uneasiness Test,

*INTT* internalization thin/low body fat, *INTM* internalization-muscular/athletic, *FP* family pressures, *PP* peer pressures, *PM* pressures media, *BIC* body image concerns, *A* avoidance, *CSM* compulsive self-monitoring, *D* depolarisation, *WP* weight phobia, *M* mouth, *FS* face shape, *TH* thighs, *L* legs, *H* harms, *MOU* moustache, *SK* skin, *BLU* blushing

**Fig. 2:**
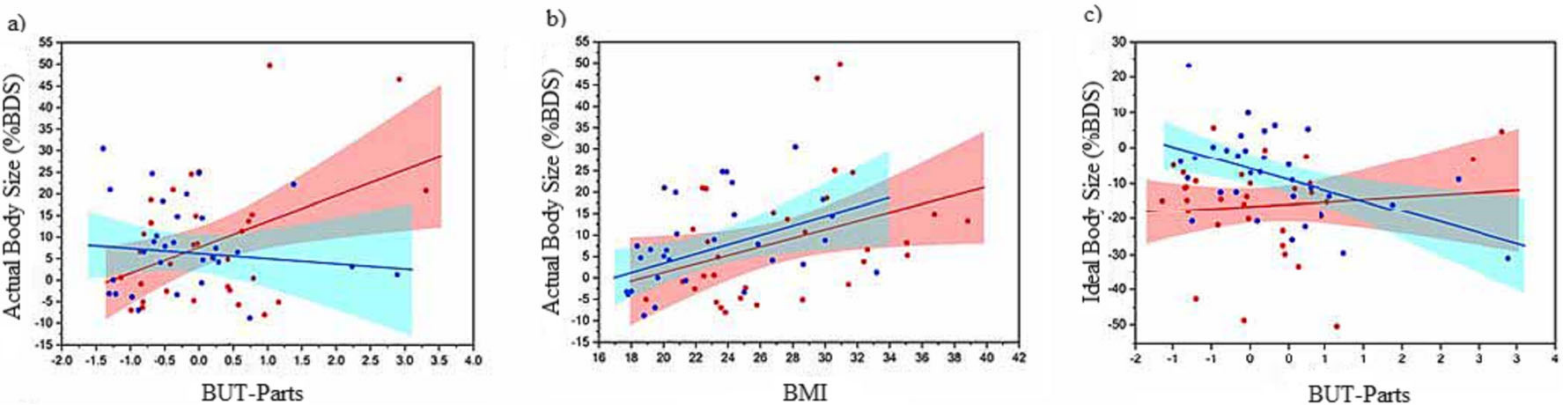
**a** Significant two-way interaction between BUT-parts and age for the perceived actual body image subcomponent. **b** Non-significant interaction between BMI and age group for the perceived actual body image. **c** Significant two-way interaction between BUT-parts and age for the ideal body image subcomponent. For all figures, the shaded regions correspond to the 95% confidence intervals for the regression slopes, which have been computed separately for each group. Blue circles with a blue regression line represent the younger women; red circles with a red regression line represent the middle-aged women

In the last step, we used PROC MIXED in SAS v9.4 to build separate linear mixed effects models of percentage distortion for perceived actual and ideal body size judgements. For each model we included putative fixed effects: age, BMI, BUT-Parts, BUT-Att, SATAQ-Press and SATAQ-Int, all of which were continuous explanatory variables. Critically, we also tested all possible two-way interaction terms. Note that for the sake of easy visualization, instead of illustrating the response surface from the statistical models as continuous 3D surface plots, consistent with the data, we plotted 2D slices through these response surfaces which show the data separated into two age groups (see Fig. 2a and b).

## Results

### Univariate statistics

Table 2 shows means and standard deviations for the demographic and psychometric questionnaire subscale scores, separately for younger and middle-aged women. The right-hand column of Table 2 shows the output of pairwise comparisons between these two groups, adjusted for multiple comparisons, using the permutation method in PROC MULTEST (SAS 262 v9.4). Middle-aged women were indeed significantly older, had higher BMIs and reported greater concerns on the thighs’ subscale of the BUT-B (this includes questions about the stomach, abdomen, hips, thighs and knees) than younger women.

**Table 2:** Demographic and psychometric responses from middle-aged (*n* = 33) women and younger women (*n* = 33)

	Middle-aged (*n* = 33) M (SD)	Younger (*n* = 32) M (SD)	Middle-aged vs. younger *p*
Age (years)	53.79 (3.72)	24.22 (4.51)	< 0.001
BMI (kg/m^2^)	27.16 (5.13)	22.74 (4.36)	< 0.005
SATAQ-4			
Internalization-thin/low body fat (max 5)	2.62 (1.03)	2.89 (0.68)	ns
Internalization-muscular/athletic (max 5)	2.16 (0.98)	2.56 (1.07)	ns
Pressures—Family (max 5)	2.08 (1.22)	1.80 (1.04)	ns
Pressures—Peers (max 5)	1.98 (1.20)	1.76 (1.02)	ns
Pressures—Media (max 5)	3.44 (1.39)	3.16 (1.33)	ns
BUT-A			
Body image concern (max 5)	1.90 (0.93)	1.45 (1.07)	ns
Avoidance (max 5)	0.87 (0.75)	0.51 (0.74)	ns
Compulsive self-monitoring (max 5)	1.36 (0.86)	1.26 (0.90)	ns
Depersonalization (max 5)	0.76 (0.75)	0.65 (0.64)	ns
Weight phobia (max 5)	2.00 (1.01)	1.55 (1.08)	ns
Global Severity Index (max 5)	1.46 (0.78)	1.13 (0.85)	ns
BUT-B			
Mouth (max 5)	1.56 (0.92)	1.26 (0.90)	ns
Face shape (max 5)	1.22 (0.82)	1.04 (0.97)	ns
Thighs (max 5)	2.79 (1.19)	1.65 (1.13)	< 0.005
Legs (max 5)	1.72 (1.21)	1.23 (1.02)	ns
Harms (max 5)	1.53 (1.08)	1.17 (0.99)	ns
Moustache (max 5)	0.86 (1.12)	0.89 (1.02)	ns
Skin (max 5)	2.02 (1.23)	1.72 (1.15)	ns
Blushing (max 5)	1.47 (1.07)	1.22 (1.00)	ns
Positive Symptom Total (max 37)	26.85 (10.56)	23.84 (12.27)	ns
Positive Distress Symptom Index (max 5)	2.29 (.75)	1.94 (.72)	ns

*BMI* body mass index, *SATAQ-4* Sociocultural Attitudes Towards Appearance Questionnaire, *BUT* Body Uneasiness Test, *ns non-significant*

Additional demographic characteristics (ethnicity, handedness and regular menstrual cycle) are reported in Table 3. We conducted a Chi-square analysis between young and middle-aged women to investigate whether there were any differences in characteristics between these two groups. There were no significant differences for ethnicity (χ^2^_1_ = 2.00; *p* = 0.157) and handedness (χ^2^_1_ = 2.60; *p* = 0.107) between groups. As expected, there was a significant difference for regular menstrual cycle (χ^2^_1_ = 32.32; *p* < .001) between groups.

**Table 3:** Demographic characteristics of middle-aged (*n* = 33) women and younger women (*n* = 32), analysed by Chi-square

	Group		
	Middle-aged *n* (%)	Young *n* (%)	Total *n* (%)
Characteristic			
Ethnicity			
Caucasian	31 (93.9)	32 (100)	63 (96.9)
Mixed race	2 (6.1)	0 (0)	2 (3.1)
Handedness			
Right	29 (87.9)	23 (71.9)	52 (80)
Left	4 (12.1)	9 (28.1)	13 (20)
Menstrual cycle			
Yes	8 (24.2)	30 (93.8)	38 (58.5)
No	25 (75.8)	2 (6.2)	27 (41.5)

### Perceived actual body size

Table 4 shows the correlation matrices between the four principal components (BUT-Parts, BUT-Att, SATAQ-Press, SATAQ-Int), age, BMI and percentage distortion for participants’ estimates of their perceived actual body size (%BDS), calculated separately for younger and middle-aged women.

**Table 4:** Pearson correlations between each of the four principal components (BUT-Parts, BUT-Att, SATAQ-press, SATAQ-Int), age, BMI and perceived actual body size distortion, presented separately for middle-aged women (*n* = 33) and younger women (*n* = 32)

		Actual	Age	BMI	BUT-Parts	BUT-Att	SATAQ-Press
Middle-aged women	Age	− 0.03	-				
	BMI	0.37*	− 0.08	-			
	BUT-Parts	0.43*	0.16	− 0.04	-		
	BUT-Att	0.39*	− 0.13	0.45*	− 0.12	-	
	SATAQ-Press	0.06	− 0.02	0.47*	− 0.00	− 0.11	-
	SATAQ-Int	0.10	0.14	− 0.09	0.03	0.13	0.09
Younger women	Age	− 0.26	-				
	BMI	0.41*	− 0.07	-			
	BUT-Parts	− 0.10	− 0.03	0.39*	-		
	BUT-Att	0.16	− 0.25	0.42*	0.08	-	
	SATAQ-Press	0.39*	0.21	0.56**	− 0.06	0.06	-
	SATAQ-Int	0.00	− 0.09	− 0.18	0.06	− 0.07	− 0.00

*p < 0*.05*, **p < 0*.005*, ***p < 0.001

*BMI* body mass index, *Att* attitudinal, *Press* pressures, *Int* internalization

For middle-aged women, percentage distortion for their perceived actual body size was significantly, positively correlated with BMI, BUT-Parts and BUT-Att, but not with SATAQ-Press or SATAQ-Int. BMI was significantly, positively correlated with BUT-Att and SATAQ-Press but not with BUT-Parts or SATAQ-Int. For the younger women, percentage distortion (%BDS) for the perceived actual body size was significantly, positively correlated with BMI and SATAQ-Press, but not with any other component. BMI was significantly, positively correlated with BUT-Parts, BUT-Att and SATAQ-Press, but not with SATAQ-Int(see Table 4).

We used PROC MIXED (SAS v9.4) to model percentage distortion for perceived actual body size. We found statistically significant main effects of BMI (*F*(1.60) = 17.19, *p* < 0.001) and BUT-Parts (*F*(1.60) = 7.31, *p* = 0.01). Critically, however, the effect of BUT-Parts was age dependent, because we found a significant interaction between age and BUT-Parts, (*F*(1.60) = 12.13, *p <* 0.001).

Figure 2a shows clearly that over-estimation of perceived actual body size increases as a function of increasing concerns about body parts in middle-aged women. Statistically, however, there is no relationship between percentage distortion and BUT-Parts for younger women, (*F*(1.28) = 3.14, *p* = .087). Moreover, since the 95% CI for their regression line includes 0% body distortion, we conclude that these groups were mostly accurate in their judgements.

Figure 2b shows plots of percentage distortion for perceived actual body size as a function of participants’ BMI, with the same colour scheme for younger and middle-aged women. Statistically, the two groups were indistinguishable and showed a significant tendency to overestimate their perceived actual body size with increasing BMI. These results suggest that distortions in perceived actual body size estimation of younger and middle-aged women are best explained by a combination of BMI, concern for body parts and the particular age group to which a participant belonged.

### Ideal body size

Table 5 shows the correlation matrices between the four principal components (BUT-Parts, BUT-Att, SATAQ-Press, SATAQ-Int), age, BMI and percentage distortion for participants’ estimates of their ideal body size, calculated separately for younger and middle-aged women.

**Table 5:** Pearson correlations between each of the four principal components (BUT-Parts, BUT-Att, SATAQ-press, SATAQ-Int), age, BMI and ideal body size distortion, presented separately for middle-aged women (*n* = 33) and younger women (*n* = 32)

		Ideal	Age	BMI	BUT-Parts	BUT-Att	SATAQ-Press
Middle-aged women	Age	0.26	-				
	BMI	− 0.11	− 0.08	-			
	BUT-Parts	0.08	0.16	− 0.04	-		
	BUT-Att	− 0.02	− 0.13	0.45*	− 0.12	-	
	SATAQ-Press	− 0.11	− 0.02	0.47*	− 0.00	− 0.11	-
	SATAQ-Int	− 0.06	0.14	− 0.09	0.03	0.13	0.09
Younger women	Age	− 0.19	-				
	BMI	0.00	− 0.07	-			
	BUT-Parts	− 0.49**	− 0.03	0.39*	-		
	BUT-Att	− 0.10	− 0.25	0.42*	0.08	-	
	SATAQ-Press	0.08	0.21	0.56**	− 0.06	0.06	-
	SATAQ-Int	0.15	− 0.09	− 0.18	0.06	− 0.07	− 0.00

*p < 0.05, **p < 0.005, ***p < 0.001

*BMI* body mass index, *Att* attitudinal, *Press* pressures, *Int* internalization

For middle-aged women, percentage distortion for their ideal body size was not significantly correlated with BMI or any principal component. BMI was significantly, positively correlated with BUT-Att and SATAQ-Press, but not with BUT-Parts or SATAQ-Int. For the younger women, percentage distortion was significantly, negatively correlated with BUT-Parts, but neither with BMI nor any other component. BMI was significantly, positively correlated with BUT-Parts, BUT-Att and SATAQ-Press, but not with SATAQ-Int(see Table 5).

We used PROC MIXED (SAS v9.4) to model percentage distortion for ideal body size. We found significant main effects of BUT-Parts (*F*(1.61) = 8.82, *p* = 0.004) and age (*F*(1.61) = 4.83, *p* = 0.03), as well as a significant interaction between age and BUT-Parts, (*F*(1.61) = 6.85, *p =* 0.01). Figure 2c clearly shows that as younger women’s body part concerns increase, their ideal body size becomes progressively slimmer. By contrast, middle-aged women selected a slimmer ideal, irrespective of their body concerns, since the regression of percentage distortion on BUT-Parts has a substantially negative intercept, together with a regression slope no different from zero, (*F*(1, 30) = 0.06, *p* = 0.816).

## Discussion

To our knowledge, this is the first study to use a personalized assessment, 2D optical distortion method, to compare young and middle-aged women’s perceptual performance of their perceived actual and ideal body image. Our analyses included also anthropometric covariates, such as BMI, body dissatisfaction and sociocultural influences, which are all factors that are well-known contributors to the aetiology and development of EDs (Culbert et al. 2015; Pedersen et al. 2018) during lifespan. Ultimately, we investigated if specific differences in negative attitudinal components of body image, i.e. beauty ideals/pressures and body-related concerns, may interact with women’ age and may affect body image perceptual self-estimates in a way which is substantially different in younger and middle-aged women. In line with a multidimensional model of body image according to which the size someone believes themselves to be is a combination of attitudinal and perceptual factors (Cash and Deagle III 1997), our results suggest that the accuracy of women’s judgements of their perceived current and ideal body shape is modulated by the age group they belong to and negative attitudes towards their bodies, particularly their concerns for body parts.

### Perceived actual body image

In agreement with studies reporting that women in the general population may overestimate their body size (Johnson et al. 2008), but in disagreement with other findings that instead suggest that women tend to underestimate their body size in the general population (Monteath and McCabe 1997; Robinson 2017), we found that middle-aged women with greater body parts concerns reported greater overestimations in the perception of their perceived actual body image. We did not observe the same outcome in younger women, who were almost accurate. Similar results were also obtained by Deeks and McCabe (2001) who reported that when middle-aged women were asked to pick a ‘silhouette’ which best corresponded to their perceived actual body size, they chose a larger figure than was objectively true. Critically and consistent with our findings, in Deeks and McCabe’s (2001) study, it was the middle-aged women who displayed higher dissatisfaction with specific body parts (lower and mid torso). As these regions are typically judged as larger than their actual size (Smeets et al. 2009) possibly due to these areas being more prone to the effects of ageing (Genazzani and Gambacciani 2006; Vanina et al. 2002), it may be plausible that overestimation of body size for middle-aged women may have occurred if focus was placed on those body parts of greater concern, when making their judgements (Kittler et al. 2007).

Both younger and middle-aged women overestimate their perceived current body size with increasing BMI, thus suggesting that as BMI increases over the lifetime, this factor continues to influence body image perceptions similarly (Holsen et al. 2012). This finding is in line with research by Wardle et al. (2006), who found that even young women with healthy BMI inaccurately overestimated their body size. Likewise, as found in Thaler et al. (2018), Tovée et al. (2003) and Zamariola et al. (2017), estimation of perceived actual body size was predicted by BMI so that women with higher BMI’s demonstrated an overestimated perception of their perceived actual body image.

A possible explanation for this finding is that body size distortion could occur as a result of an individual’s real body weight, as well as societal pressures to obtain a thin body size. Particularly women with higher BMI’s may have had greater discrepancies in their ability to estimate their own body size, as they may perceive their body to be significantly larger than what society classes as thin, which as a result may distort their own mental image of the self (Arciszewski et al. 2012; Zamariola et al. 2017). Societal stigmatization of greater weight may have also fed into body image concerns for these women, which has been previously associated with overestimations of body size (Thaler et al. 2018).

An additional explanation could be that as larger body sizes are more typical in middle-aged women of Western societies (Sowers et al. 2007), this may have impacted and altered perceptions of a body silhouette classed as the ‘norm’, compared with a body size classed as being overweight (Robinson 2017; Robinson and Kirkham 2014).

Indeed, according to the ‘Social Comparison Theory’, individuals make constantly evaluations about physical characteristics, such as body size by looking at the appearance of those around us, which in turn may provide an internal standard (norm) or internal representation of what is normal (Festinger 1954; Mussweiler 2003). With these regards, the on-going obesity epidemic in both non-developing and developing countries might have led to a recalibration of body shape and particular to a perception that larger body sizes are considered ‘normal’. If this was the case, then this altered shift in standard models of different BMI classifications may have caused an overestimated shift in perceived actual body size estimations, if middle-aged women used these standard models to base their judgements on their own body.

Nevertheless, for younger women only, we found a positive association with sociocultural influences, i.e. pressures from the media, family and peers (SATAQ-Press) with an increase in perceived actual body image distortions. This is in line with previous research reporting that although middle-aged women still care for their bodily appearance, they are less influenced from societal pressures compared with younger women, who are more influenced by these pressures (Pruis and Janowsky 2010; Lewis and Cachelin 2001). This could be due to differences concerning traditional (television) and social media exposure (Facebook, Instagram), with younger women having more exposure than middle-aged women (Baugh 2009; Wadsworth and Johnson 2008).

### Ideal body image

In the present study, both younger and middle-aged women consistently preferred a slimmer body size when asked to judge how they would like to appear, a result which is largely in agreement with the current literature (Baugh 2009; Lewis and Cachelin 2001; McCabe et al. 2007; Pruis and Janowsky 2010). Interestingly, as younger women’s body part concerns increase, their ideal body becomes progressively slimmer. By contrast, middle-aged women selected a slimmer ideal body, irrespective of their body concerns.

One possible explanation for this finding is that younger women may have placed a greater importance for attractiveness on specific body parts, which prior research has found to be in the lower region of the body, i.e. stomach and thighs (Irvine et al. 2019; Stanford and McCabe 2002; Ralph-Nearman et al. 2019). If young women believe that their body parts are not similar to what they perceive to be attractive in terms of size, then their desire to be thinner will be greater (Stanford and McCabe 2002). Furthermore, ‘thinspiration’, a class of body-idealizing content that currently has emerged on social media, seems to be more important for younger females, leading to young women to generally compare various body parts of the ‘ideal model’ to their own (Griffiths et al. 2018).

For both younger and middle-aged women, there was no effect of BMI on their ideal body image perceptual self-estimates. This is somehow surprising since it was expected, particularly for the middle-aged women, a relationship with higher BMI and ideal body image distortions, as middle-aged women were also those women who showed greater distortion in their perceived actual body size. Also, previous research has emphasized how BMI can account for body dissatisfaction in middle-aged women (Ålgars et al. 2009; Dunkel et al. 2010; McKinley and Lyon 2008) which results in a greater drive for thinness (Keski-Rahkonen et al. 2005; Lewis and Cachelin 2001). Instead, our findings are in line with Pruis and Janowsky (2010) in that BMI was not a predictor of ideal body image in older women. In addition, it offers support for findings of Cheung et al. (2011), in that majority of women with healthy BMI still have a desire for a slimmer body physique. Therefore, it is not just women with higher BMIs who have a greater desire for a slimmer ideal body but also females with normal range BMIs. Moreover, as suggested by Cheung et al. (2011), it could be plausible that the ideal body image is more influenced by factors such as body self-esteem, which contribute towards body dissatisfaction, and that BMI is less important in influencing an individual’s ideal body image.

### Limitations

Certain limitations of the present study should also be acknowledged.

First, although the BIR software has been proved to be successful in investigating perceptual body image in healthy and ED populations (see Cazzato et al. 2014, 2016; Zamariola et al. 2017), and is ecologically valid in the sense that it resembles a person’s mirror image, nevertheless the programme does not adjust an individual’s arms or face. Therefore, particularly at the extremes of thinness and fatness, there may be image distortions—i.e. departures from an ecologically valid image—which may cause participants to adopt a compensatory strategy, whereby participants’ judgements of the apparent body size of the person in the stimulus might be based on the computation of surface area, or perhaps perimeter-area ratio. Yet, we believe that it is unlikely that the BIR inability of altering the face and/or the arms of participants might have affected differently the two samples of women, given that both groups displayed (low) similar levels of concerns for such body parts (as measured by the BUT-B). Nevertheless, we believe that keeping the participants’ face during the perceptual tasks might have added strength to the individualized assessment procedure, as it might have improved the ecological validity of the test and reinforced women’s self-body identification during task performance.

Yet, it would be beneficial in future studies to investigate body image perception using stimulus images that do not have the limitations listed above. For example, in previous research of Cornelissen et al. (2017), different 3D avatars were generated depicting realistic BMI physiques. As well as more realistic 3D representations (see Keizer et al. 2016, for a clinical application of full body VR in EDs), this software should enable all body parts to be adjusted so as to represent a more accurate reflection of varying body sizes and that individuals can view more than just a frontal perspective. This is especially important considering the natural occurring age-related changes in older women which result in different body composition and fat distribution than younger women (Genazzani and Gambacciani 2006; Hughes et al. 2004).

With these regards, a recent study from Ralph-Nearman et al. (2019) has tested the feasibility of a novel mobile tool, the so-called Somatomap, that allows individuals to visually represent their perception of body part sizes and shapes, as well as areas of body concerns and record the emotional valence of concerns. In light of the results of our current study which highlight the importance of addressing specific body part concerns in women and related visual size (mis)perceptions, it would be extremely useful to adopt a tool with such properties when assessing multiple components of body image across lifespan in future.

Second, although previous studies of Cornelissen and co-authors(Cornelissen et al. 2015, 2017; Irvine et al., 2018) demonstrated that attitudinal components of body image can be compressed into a *single* principal component reflecting variation in attitudes to body shape, weight and eating, self-esteem and tendency to depression, yet in this study we did not include a measure of self-esteem and depression which could have mediated the need to appear thinner. With these regards, albeit no evidence for a specific role of self-esteem has been reported when investigating specific age-related differences in perceptual body image in previous investigations, yet it would be interesting to investigate in the future the link between self-esteem and body image concerns (Stapleton et al. 2017), as well as repeated dieting behaviours in older women.

## Conclusions

Despite the limitations discussed, the present study provided, for the first time, evidence that performance at tasks that measure perceptual and attitudinal components of body image are essentially different in young and middle-aged women. Most importantly, we have demonstrated that distortions in perceived actual and ideal body size estimation of younger and middle-aged women are best explained by a combination of BMI, concern for body parts and the particular age group to which a participant belonged.

Overall, these results suggest that women regardless of age show perceptual and attitudinal body image distortions, yet it is important to focus on specific concerns towards body parts, which accounts for perceived actual body image perceptions for middle-aged women and a desire to appear slimmer for young women. Thus, this study highlights the need for a multidimensional and personalized computerized approach for studies of body image in women across lifespan, which includes women of a variety of ages and a multitude of potential attitudinal factors of body image, as well as women’s perceptions and concerns of specific body areas.

## Electronic supplementary material

ESM 1 (DOCX 14 kb)

ESM 2 (DOCX 18 kb)

## Data Availability

The datasets analysed during the current study are not publicly available due lacking participant consent for data sharing with third parties (according to our current General Data Protection Regulation, GDPR) but are available from the corresponding author on reasonable request.
