# Supplementary material for: The ageing body: contributing attitudinal factors towards perceptual body size estimates in younger and middle-aged women
Source: Arch Womens Ment Health. 2020 Jun 19;24(1):93–105. doi: 10.1007/s00737-020-01046-8 (PMC7929965; doi:10.1007/s00737-020-01046-8)
Supplement: Supplementary file 1 — (DOCX 14 kb) [file 737_2020_1046_MOESM1_ESM.docx]

**Electronic Supplemental Materials 1**

**Body Image Revelear (BIR)**

The BIR provides a measure of the discrepancy between the dimensions of the real image and the sizes attributed by the participant during the task. It works by importing a participant’ real picture into the software, with the experimenter selecting the parts that would be modified, that is from the neck to the feet. The BIR employs an algorithm of two-dimensional modelling with a morphing/warping technique which reflects the real pattern of weight loss and gain in women and men. This method is useful and precise because considers the body image (simultaneously the whole body and parts of it) and its proportions, giving an extremely realistic visualisation. To this aim, a standard distortion curve has been obtained by originally sampling anthropometric data and simulative/emulative curves of about 40 Caucasian and African-American males and females. This technique is preferable to existing techniques such as video-distortion using anamorphic lenses, in which for e.g., the morphing is based on the division into frames of images or other software that examines the whole picture, and which does not take into account the body shape (entire or its parts), which is instead taken into account by the BIR. Furthermore, to test the validity and reliability of the BIR software, data from a large sample of 540 individuals were analysed. The data of healthy participants have been also compared with data those collected on patients with eating disorders (N = 67), confirming good psychometric properties. Ultimately, the BIR allows different body image subcomponents to be evaluated and correlates well with self-report measures of body image disturbances (see for e.g., Cazzato et al., 2014, 2016; Zamariola et al., 2017).

During the experiment, all stimuli were displayed on a 15.6‐inch LCD monitor (resolution, 1,024 × 768 pixels; refresh frequency, 60 Hz), controlled by the BIR software running on a Windows PC. During each trial, the stimulus appeared in the middle of the screen, on a white background, so that the stimulus body subtended a visual angle of 13.5° vertically × 17.94° horizontally. At the beginning of each trial, the picture was shown with a maximal level of distortion at the lower (slimmer) or upper (larger) extreme. A value of 0% indicates the original model’s size, negative values indicate a distortion toward a slimmer figure, and positive values indicate a distortion toward a larger figure, with respect to the actual size of the model (see Fig. 1). Hence, there were no limits to the way participants could adjust their picture i.e. they could press the key as many times as they wish or alternate between pressing the plus or minus key and no time constraints in responding. A total of 24 trials were presented for the actual and ideal body size tasks (12 trials respectively). The order of the questions within the two current and ideal tasks was balanced according to a Latin square procedure (ABBA). Furthermore, the initial distortion level of the image presented at the beginning of each trial (either−50 % slimmer or +50 %larger) was randomized in each block, for each task. The dependent variable was the difference between the models’ real size and the participants’ responses to the perceived actual/ideal body shape tasks. The value (as a percentage) corresponding to the final level of distortion for the body (body distortion score, BDS%) was automatically saved on the computer for offline analysis.
