# Supplementary material for: The ageing body: contributing attitudinal factors towards perceptual body size estimates in younger and middle-aged women
Source: Arch Womens Ment Health. 2020 Jun 19;24(1):93–105. doi: 10.1007/s00737-020-01046-8 (PMC7929965; doi:10.1007/s00737-020-01046-8)
Supplement: Supplementary file 2 — (DOCX 18 kb) [file 737_2020_1046_MOESM2_ESM.docx]

**Electronic Supplemental Materials 2**

**Correlation and Principal Component Analysis (PCA)**

Given the substantial inter-correlations within and between BUT-A, BUT-B, and SATAQ-4 psychometric tasks, we used PROC FACTOR (SAS v9.4) to run a Principal Components Analysis (PCA) with Varimax (orthogonal) rotation on the 18 psychometric subscales. The Kaiser-Meyer-Olkin (KMO) measure of sampling adequacy (which indicates the degree of diffusion in the pattern of correlations) was 0.86 suggesting an acceptable sample. Four principal components had eigen values greater than Kaiser’s criterion of 1 (i.e., 8.84, 1.69, 1.52, and 1.12) which, together, explained 73% of the variance. The scree plot showed an inflexion, i.e. Cattel’s criterion, which also justified retaining just four principal components. The residuals were all small, and the overall root mean square off-diagonal residual was 0.051, indicating that the factor structure explained most of the correlations. Principal component 1 (henceforth called BUT-Parts) had high loadings on the BUT-B subcomponents: harms, face shape, legs, blushing, mouth, thighs, moustache and skin. Principal component 2 (henceforth called BUT-Att) had high loadings on the BUT-A subcomponents: weight phobia, compulsive self-monitoring, body image concern, avoidance and depolarisation. Principal component 3 (henceforth called SATAQ-Press) largely accounted for SATAQ pressures, with high loadings from family, peers and media. Principal component 4 (henceforth called SATAQ-Int) largely accounted for SATAQ internalisation with high loadings from thinness and muscularity. The rotated factor loadings are displayed in Table 1 below. Factor loadings less than 0.40 have been excluded for clarity.

**Table 1.** Rotated factor loadings from the Principal Component Analysis (PCA) of psychometric tasks. Factor loadings < .40 are not displayed.

|  | PC1  BUT-Parts | PC2  BUT-Att | PC3  SATAQ-Press | PC4  SATAQ-Int |
| --- | --- | --- | --- | --- |
| BUT-Harms | .83 |  |  |  |
| BUT-Face shape | .83 |  |  |  |
| BUT-Legs | .78 |  |  |  |
| BUT-Blushing | .76 |  |  |  |
| BUT-Mouth | .74 |  |  |  |
| BUT-Moustache | .61 |  |  |  |
| BUT-Skin | .61 |  |  |  |
| BUT-Thighs | .60 |  | .44 |  |
| BUT-Avoidance |  | .82 |  |  |
| BUT-Weight Phobia | .48 | .80 |  |  |
| BUT-Body Image Concern |  | .78 |  |  |
| BUT-Depolarisation |  | .71 |  |  |
| BUT-Compulsive Self-Monitoring | .48 | .64 |  | .42 |
| SATAQ-Family Pressures |  |  | .88 |  |
| SATAQ-Pressures Peers |  |  | .69 | .49 |
| SATAQ-Pressures Media |  |  | .53 |  |
| SATAQ-Internalisation- Muscular/athletic |  |  |  | .89 |
| SATAQ-Internalisation-Thin/low body fat |  |  |  | .69 |

*Note: BMI= Body Mass Index; SATAQ-4= Sociocultural Attitudes Towards Appearance Questionnaire; BUT= Body Uneasiness Test*
